# International Medical Congress at Vienna

**Published:** 1873

**Authors:** 


### INTERNATIONAL MEDICAL CONGRESS AT VIENNA.

The third meeting of this body was opened on September 1st, at Vienna, by Archduke Rainer. The questions discussed were Vaccination, Prostitution, Cholera and Quarantine, and Sewage.

On vaccination the following abstract embaces the principal results:

Dr. Kaposi read the report and the questions of detail connected with it, the most important of which was, Shall there be vaccination or not? The speaker pleaded for vaccination, and wished to see it introduced by law. The proportion of mortality was undoubtedly greatly in favor of the vaccinated. According to experience the proportion with vaccinated persons was between 0 per cent.—11.5 per cent., *i. e.*, in the average four per cent.; with persons not vaccinated between 14.5 per cent.—60.6 per cent.; therefore, in the average thirty per cent. The next question, important also for the public in general, was in what time of the year the vaccination should take place? The answer was given that the season of the year had no influence on vaccination; at the same time the speaker modified his idea that children ought to be vaccinated as early as possible; a child, only four or five days old, could be vaccinated without the least danger.

Dr. Neuman brought out some interesting statistical notes in favor of vaccination. In small-pox epidemics mortality is notoriously five times greater with those who have not been vaccinated than with those who have been vaccinated. During the last epidemic in Berlin forty per cent. of the non-vaccinated, two of the vaccinated, and five-tenths per cent. of the revaccinated died. [Applause].

Mr. Knopfl (Siebenburgen) called the vaccination question "a new miscarriage of statistics built on sand." [Great hilarity.]

Dr. Kaposi made the final speech, and said that the moral decision about the first question, "Shall there be vaccination or not?" had been given, and that it was now the duty of the Congress to express their opinion honestly and openly.

With regard to the importance of this principal question, Prof. Hebra proposed a resolution, about which the members of the Congress should vote by ballot, signed in their own handwriting.

The resolution was:

"The third International Medical Congress declares the cow-pox vaccination necessary, and recommends to the respective Governments the introduction of obligatory vaccination."

One hundred and sixty-two ballots had been given, out of which 155 were for and 5 against the resolution. On one ticket there was no name, and on another ticket the name was illegible.

Regarding syphilis and prostitution, an outline of an international legislative enactment was agreed on, providing that medical treatment of syphilis should be carried on under the direction of the authorities, who should choose the medical officers for the purpose, undertaking also the cost when necessary; that special wards for the treatment of syphilis should be instituted in all hospitals, also under the direction of the public authorities; and that all candidates for licenses to practice medicine should undergo a special examination on syphilis.

The discussion on quarantine in cholera led to the adoption of resolutions that land and river quarantine ought to be abolished, but that maritime quarantine should for the present be continued; and that an international committee should be formed to study the means by which cholera is spread, and to frame regulations more efficient than those at present in force. Regarding quarantine in general, it was agreed that it ought to be limited to the time necessary for the disinfection of the ship, crew, and passengers. It was also recommended that a permanent international committee should be appointed to determine what diseases of men and animals should be subject to quarantine, and to draw up a plan for universal application.

A rather long series of resolutions were passed with regard to the drainage of towns, concluding with the following: "All towns should be obliged to take into mature consideration, with the assistance of approved experts, all questions regarding the cleansing and keeping clean of the ground of the town, and the disposal of ordure. This is required in the interest both of the inhabitants and of national economy in the widest sense of the word."

A resolution recognizing the necessity of an International Pharmacopœia was passed. It was agreed that the Pharmacopœia should contain the most important and generally recognized remedies, and their most necessary excipients and corrigents, with a sufficient scientific description; and that the metric system should be adopted. It was also agreed that it should be recommended to the next International Congress to organize a committee for the formation of such a Pharmacopœia.

Brussels was chosen as the place for holding the next International Medical Congress in 1875.

The session was brought to a close with an address by Professor Rokitansky.—*Medical and Surgical Reporter.*

